# Supplementary figures and images for: Epithelial Cell Line Derived from Endometriotic Lesion Mimics Macrophage Nervous Mechanism of Pain Generation on Proteome and Metabolome Levels
Source: Biomolecules. 2021 Aug 17;11(8):1230. doi: 10.3390/biom11081230 (PMC8393596; doi:10.3390/biom11081230)

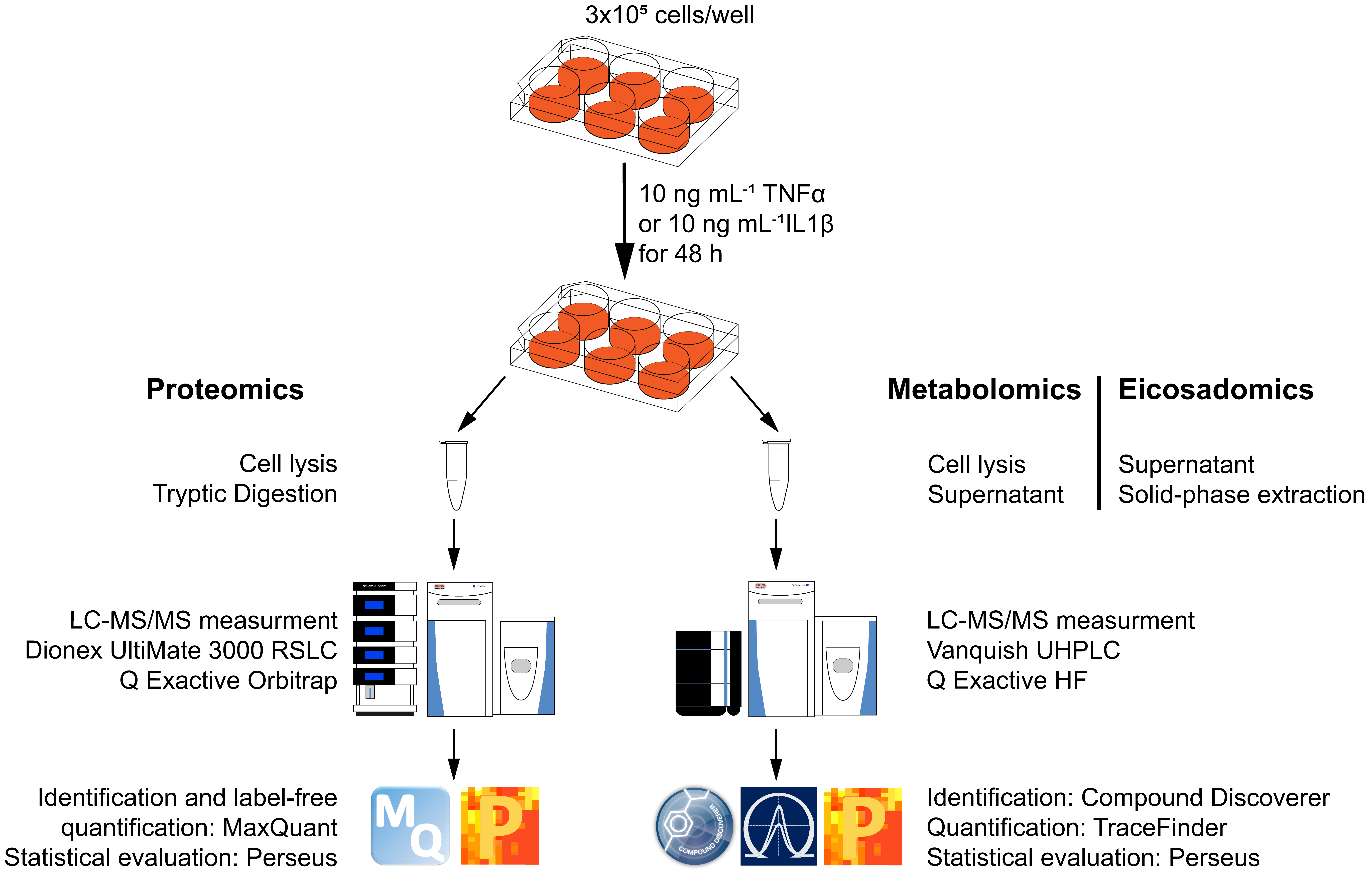

Supplement: Supplementary file 1 [file biomolecules-11-01230-s001.zip › Suppl-Figure_01.tif]

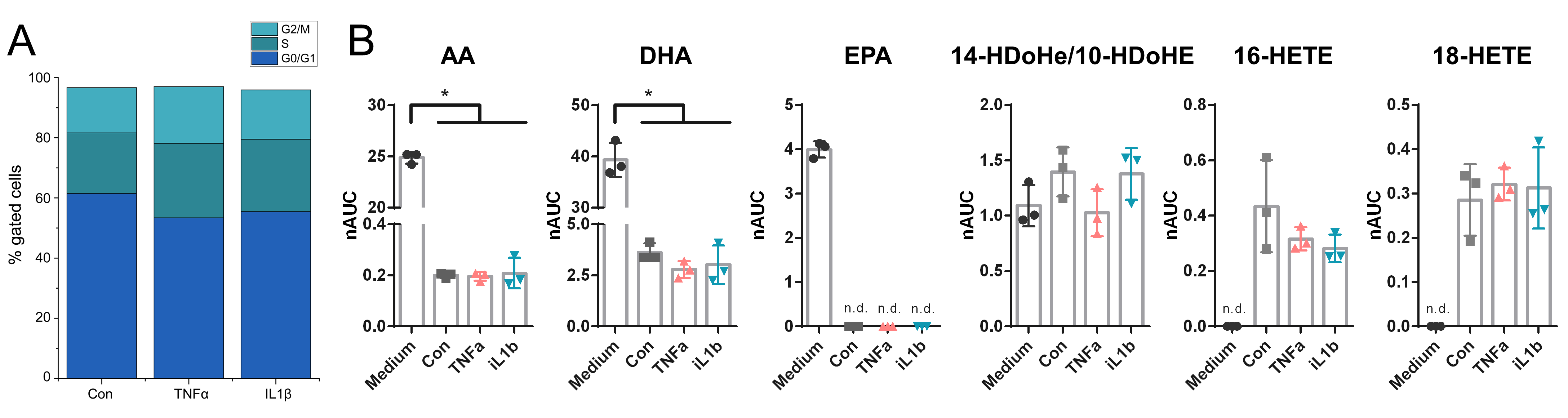

Supplement: Supplementary file 1 [file biomolecules-11-01230-s001.zip › Suppl-Figure_02.tif]

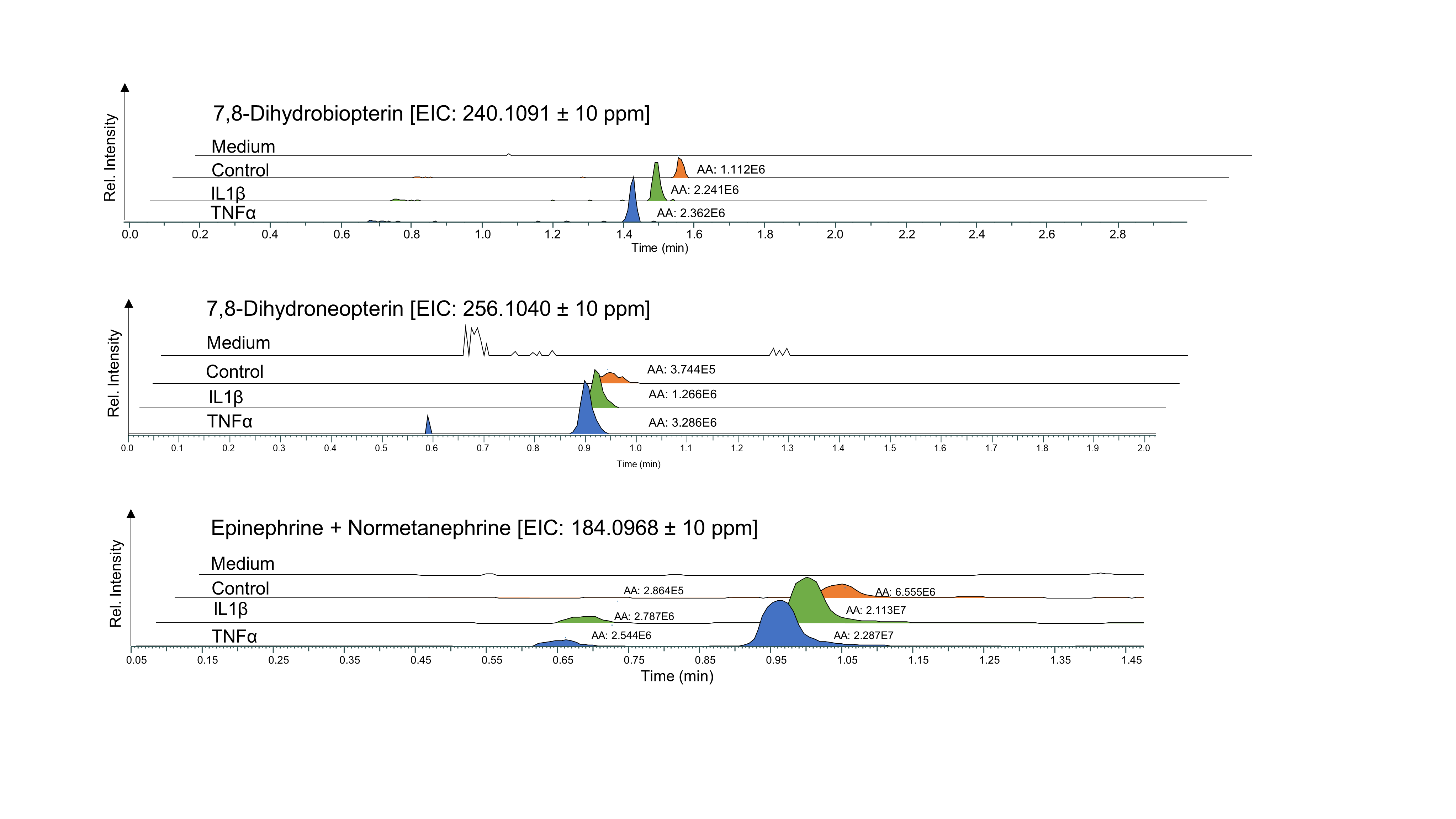

Supplement: Supplementary file 1 [file biomolecules-11-01230-s001.zip › Suppl-Figure_03.tif]
